# Effect of Interlaminar Toughness on the Residual Compressive Capacity of Carbon Fiber Laminates with Different Types of Delamination

**DOI:** 10.3390/polym14173560

**Published:** 2022-08-29

**Authors:** Yao Zhang, Deng’an Cai, Yanpeng Hu, Nan Zhang, Jinfeng Peng

**Affiliations:** State Key Laboratory of Mechanics and Control of Mechanical Structures, Nanjing University of Aeronautics and Astronautics, Nanjing 210016, China

**Keywords:** delamination defect, interlaminar toughness, digital image correlation, magnetic guidance, steel cylindrical particle, compression

## Abstract

In this paper, the effect of interlaminar properties and the type of delamination defects on the residual compression properties of carbon fiber laminates were experimentally investigated. A new method, which employed magnetic force to guide the arrangement direction of stainless steel particles between layers of laminates, was adopted to improve the interlayer toughness. The digital image correlation, C-scan, and micro-CT were used to measure and identify the compression failure damages. Test results showed that the compressive strength of the intact carbon fiber laminates was 299.37 MPa, and the one of specimens containing the deeply buried delamination, the through-width delamination, and the surface delamination decreased by 55.98 MPa, 58.69 MPa, and 60.23 MPa, respectively. The compressive strength of the specimens containing the deeply buried delamination only decreased by 14.01 MPa when the mode I toughness increased by 81.88%, and the specimen containing the surface delamination only decreased by 30.86 MPa when the mode II fracture toughness increased by 87.72%. However, improving the fracture toughness could not strengthen the specimens containing the through-width delamination. Moreover, a qualitative dynamic damage relationship, which described the relationship between delamination expansion and compression damage vividly, was proposed. The reason the increase of the toughness could improve the residual compression performance of the laminates containing delamination was that the higher fracture toughness hindered the secondary expansion of the delamination during the compression process so that the delamination area could almost remain unchanged.

## 1. Introduction

Carbon fiber laminate is a new material with high specific strength and high specific modulus, and thus widely used in various fields [1,2,3]. Delamination damage of composite materials usually refers to an interlaminar matrix cracking phenomenon distinguished from in-plane fiber and matrix damages. Since the interlayer is often a relatively fragile matrix-rich area, the susceptibility to delamination has become the most common and critical drawback of carbon fiber laminated materials [4,5,6,7,8,9,10,11,12,13].

Why delamination damage is so fatal is attributed to the unique structure of laminated composite materials [14,15]. It is known that the rigorous lay-up design is an important factor to achieve excellent mechanical properties of laminates. Once the material is delaminated over a large area, the interrelationship between the layers is broken, the material is divided into several thin sub-layers of material along the thickness direction, and thus the original lay-up design is altered, which may result in a more complex stress state within the sub-layers. Therefore, it is easy to produce local failure and reduce the overall performance of the material, especially the compressive strength of the laminates. The delamination of laminated materials is mainly related to the layup sequence, the properties of raw materials, temperature, and strain rate [16,17].

The most common way to study the residual properties of laminates is the compression after the impact test [18,19,20,21]. This method can evaluate the impact resistance of materials by their residual compression properties after being impacted under different impact energies. The impact damage types range from barely visible damage to penetration damage depending on the impact energy. During the impact process, the impacted point is depressed by the punch pressure and serious local bending occurs. The bending process produces interlayer shear stress, the main cause of the delamination of the laminates [22,23,24,25,26,27]. Therefore, the compression after the low-energy impact test is actually to study the delamination resistance and residual properties of materials containing delamination defects. However, the damage introduced by this method needs to be detected by other instruments to ensure that neither is the fiber broken nor is the matrix cracked by impact. It is very difficult to accurately introduce delamination damage alone by means of impact.

To study the influence of delamination alone on the residual compressive capacity of the laminates, therefore, some scholars artificially made the initial delamination during the molding of composite materials [14,28,29,30,31]. The method of embedding delamination in the specimen is similar to the one used for measuring interlaminar fracture toughness, such as the one in the double cantilever beam (DCB) test and the end notched fracture (ENF) test [32,33,34]. Through this method, not only can the size and shape of the initial defect be changed artificially, but also its location can be adjusted conveniently.

The delamination of composite laminates is one of the factors limiting their practical application. Thoroughly understanding the mechanical behavior of delaminated composites is important for damage tolerance design of laminated composite structures. Currently, scholars have studied the effects of delamination location [14,35] and delamination size [35,36] on the residual material properties. However, it is rare to study the influence of delamination type and interlayer toughening on the residual properties of laminates with delamination. Different types of delamination defects may affect the location of damage initiation, the form of crack propagation, and the characteristics of local deformation of the material during compression due to different boundary conditions and spatial locations. Since the damage of laminates is often accompanied by delamination, an increase in fracture toughness may indirectly affect the compressive properties of the material and increase its damage tolerance. Therefore, the objective of this paper is to investigate the effect of interlaminar toughness on the residual compressive properties of composites with delamination defects. The toughening effect can be achieved by filling other materials in the interlayer. Many studies have shown that the particles of different sizes and materials can play a role in improving the properties of all aspects, including the interlayer properties. It is also an important topic in the development of laminated composite materials [37,38,39].

The residual compressive properties of the specimens containing three different types of delamination defects are tested and compared, and a new interlayer toughening method is used to toughen the materials. Magnetic force is used to guide the cylindrical 410 stainless steel particles aligned along thickness direction to achieve the interlayer toughening. This toughening method is able to guide the orientation of the particles arranged between the layers from the outside of the laminate during the forming process. The particles uniformly arranged along the thickness direction can form a stronger bridging structure than randomly distributed particles, thus enhancing the fracture toughness of the material more effectively. By comparing the compressive properties of materials with and without a toughening treatment, the effect of interlayer toughening on the residual properties of materials with delamination is obtained. Furthermore, the mechanical behavior of different types of specimens under compression is studied and the mechanism of toughening is discussed. Based on the test results, a dynamic damage relationship suitable for describing the relationship between delamination propagation and compression failure is proposed. It is hoped that the reported results can provide a reference for the innovative design of interlaminar toughening of composites and methods to improve the material damage residual properties.

## 2. Experiment

### 2.1. Specimens Preparation

**Figure 1:**
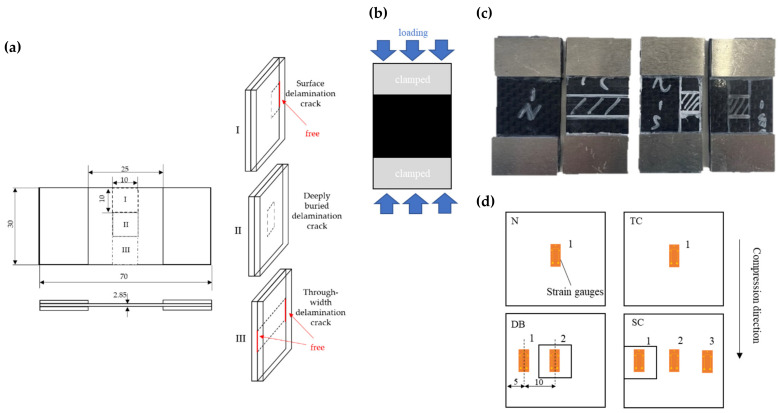
Schematic diagrams of (**a**) specimens with different types of embedded delamination; (**b**) loading method; (**c**) physical drawing of specimens; (**d**) location of strain gauges.

A compression specimen was designed, as shown in Figure 1. The selected material was 6 k bi-directional plain carbon fiber/epoxy (T300/E51) and the stacking sequence was [(0, 90)]_6_. The thickness of the specimen was 2.85 mm. Aluminum tabs were pasted at both ends of the specimens, schematically shown in Figure 1a.

The wet hand layup molding was adopted to fabricate the specimens. Although the molding method is a primitive method, it is suitable for artificially introducing delamination between layers and particles. To artificially introduce delamination defect, a piece of 0.04 mm thick poly tetra fluoroethylene (PTFE) film was placed in the designated location between ply-3 and ply-4 to prevent resin infiltration. Three types of embedded delamination were introduced, namely, the deeply buried delamination crack (DB), the surface delamination crack (SC), and the through-width delamination crack (TC), as shown in Figure 1a,b. In addition, the letter N was used to indicate the type of specimens without embedded delamination.

The interlayer toughening method used is a new type of magnetically guided particle toughening method that uses a magnetic field to guide the distribution direction of the particles filled in the interlayer to enhance the particle toughening effect. To ensure that the particles could be magnetically guided, the 410 stainless steel particles were chosen as the filler. The shape of the particle is cylinder with a cross-sectional radius of 0.015 mm and a length between 0.10 and 0.20 mm. A steel particle with such a size is more conducive to the rotation of the particles between the layers, and can enter the pores of the fiber cloth to make the coupling between the fibers and the layers stronger. Before the composite material was laid, a certain mass of particles was weighed by an electronic scale with an accuracy of 0.1 mg. Particle density mentioned here and elsewhere in this article denotes the content of particles per unit area between layers and its unit is g/m^2^, which is different from the material intrinsic density. Three different densities of particles were prepared, namely, 0 g/m^2^ (particle-free), 30 g/m^2^, and 60 g/m^2^, respectively. For the convenience in presentation, symbols 0, 30, and 60 were used to distinguish the particle densities in different groups of specimens.

To fill the particles into layer, a 100-mesh sieve was used to put the particles between the layers. In the preparation of the specimens for this experiment, the particles were only filled into the layer containing the delamination defect. One more step for manufacturing certain specimens, named as SZ group, is to apply a fixed direction magnetic field from the outside when the resin has good fluidity. The reason for this step is to effectively guide the cylindrical particles to be distributed vertically. For comparison, samples with the same particle density without magnetic field guidance were also prepared, named as SJ group. In order to distinguish different kinds of specimens more conveniently, the rule to name the specimens is “Density symbol”—“Processing method”—“Crack type”. For example, a specimen, containing an embedded surface crack, with a particle density of 30 g/m^2^ and guided by a magnetic field was named as “30-SZ-SC”.

**Figure 2:**
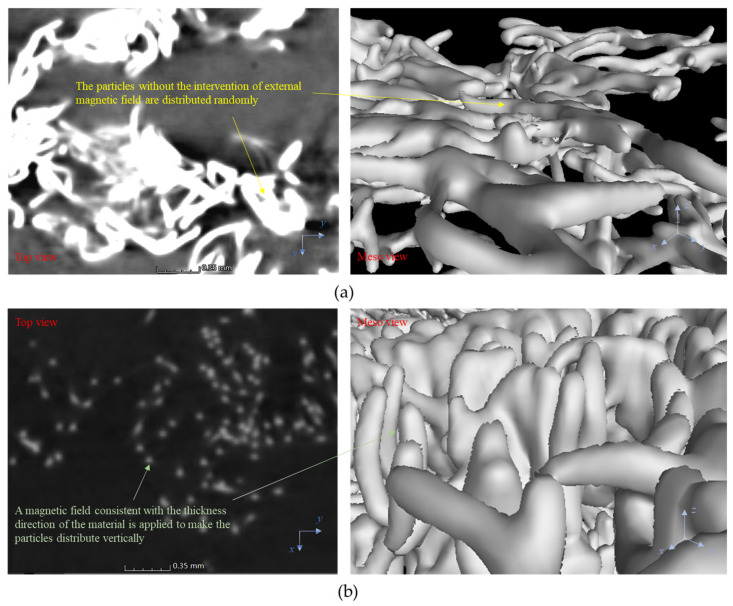
Comparison of micro particle distribution diagram of (**a**) SJ and (**b**) SZ specimens.

In order to confirm that the magnetic guidance method can indeed change the distribution direction of particles, the article also scans the interior of the two materials with the micro-CT technology, as shown in Figure 2. From the top view (the micro particle distribution after local matrix removal), it can be seen that the magnetic guidance has an obvious influence on the distribution direction of particles, which leads to obvious differences in the interlayer micro structure of the two types of specimens.

In ASTM D7137 standard [40], the size of the specimen required by the traditional test method makes it be difficult to accurately position the artificial delamination defect, and to ensure the film is not squeezed to other locations during the long forming operation time. It should be also noted that the width size of the specimen could not be too small, because the wider width can better reflect the characteristics of different kinds of delamination defects and make the specific influence more obvious in the compression process. Therefore, the specimen width is increased to 30 mm in this work. According to the thickness reference and formula provided in standard ASTM 3410 [41], the thickness of the specimen should be at least 2.10 mm to prevent the occurrence of Euler (column) buckling. Apparently, the thickness with 2.85 mm of the specimen meets the requirement. Since the mechanical behavior of the material may be altered due to the embedded delamination and toughening operations, it is still necessary to monitor the deformation of the specimen during compression with the strain gauges. The strain gauge locations for each type of specimen are shown in Figure 1d.

### 2.2. Experimental Methodology

**Figure 3:**
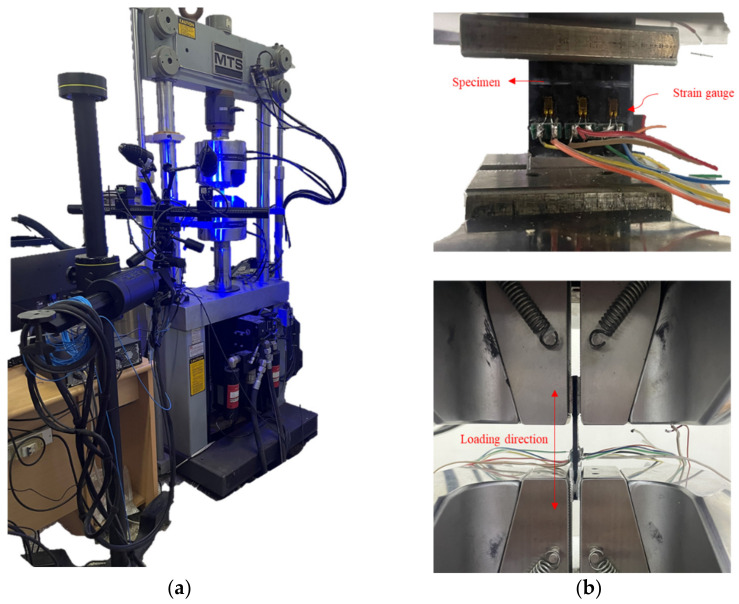
Quasi-static compression tests: (**a**) DIC and MTS 810.25 tester; (**b**) strain gauges and clamps.

The quasi-static compression test was completed by the MTS 810.25 tester and the digital image correlation (DIC). The specimen was installed to ensure that its long axis was parallel to the loading direction to prevent shear stresses, as shown in Figure 3. Displacement loading mode was used. During the test, the strain, load, and displacement data were recorded until the specimen showed obvious fracture or local instability or a serious delamination expansion, which caused a rapid drop of the applied load.

### 2.3. Damage Identification and Mechanical Response Measuring Equipment

#### 2.3.1. C-Scan Damage Identification

**Figure 4:**
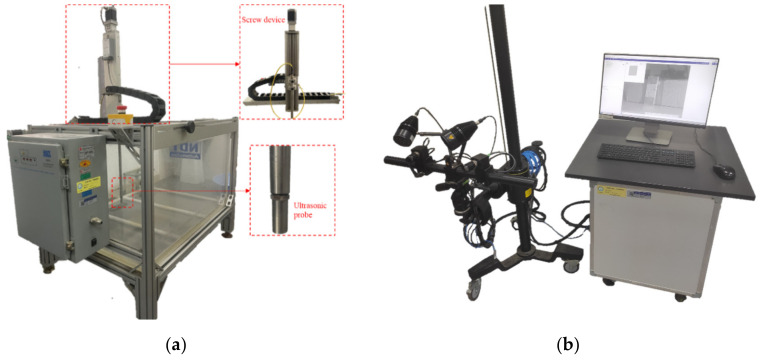
Equipment: (**a**) UltraPAC ultrasonic C-scan system; (**b**) DIC measurement system.

The UltraPAC ultrasonic C-scanner, shown in Figure 4a, is used to identify the initial and final delamination defects. It consists of a 15 MHz ultrasonic probe; a signal acquisition device; a filament guide system that allows the probe to move freely in X, Y, and Z directions; a reservoir; and a computer with installed test software. The layer containing defects is found by using the return time difference of sound waves between different layers in the composite, and the damage range map is drawn by detecting the difference of signal intensity reflected by the delamination area and the intact area, so as to reflect the change of delamination state before and after test.

#### 2.3.2. Digital Image Correlation (DIC)

To monitor the global displacement changes in all directions during the compression process of the specimen, especially the distribution of out-of-plane displacement, the DIC test technology was employed to observe and measure the global compression process of the specimens. The measurement hardware and software (ARAMIS provided by GOM, Leipheim, Germany), shown in Figure 4b, are produced by GOM Company in Germany. Before measurement, the uniform and dense speckle on the surface of the specimen were sprayed in advance, and the DIC equipment was calibrated. The main components of the measurement equipment included lens, light source, movable sliding rod, and support. The double lens is used to capture the position of the speckle and form spatial curved surface components. The change of the relative position of speckle was compared by taking photos to calculate the deformation of the specimen surface.

### 2.4. Interlaminar Fracture Toughness Test

**Figure 5:**
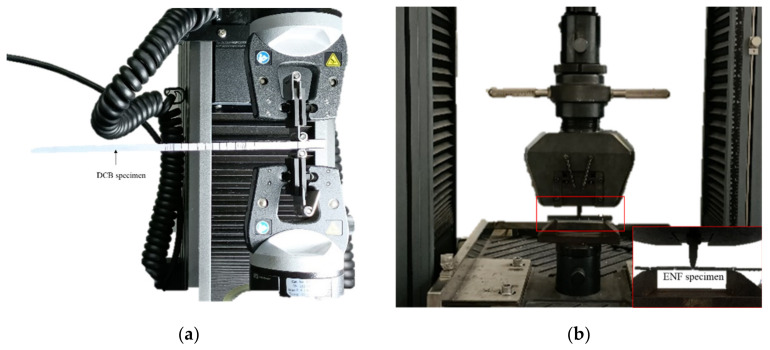
Fracture toughness tests: (**a**) DCB test and (**b**) ENF test.

To study the influence of toughening methods on the fracture toughness of materials, the interlaminar mode I and mode II fracture toughness of materials were also tested under different working conditions. As shown in the Figure 5, the DCB and ENF tests were conducted, respectively referring to ASTM D5528 and ASTM D7905 [42,43,44,45].

## 3. Results and Discussion

### 3.1. Comparison and Discussion of Experimental Results

#### 3.1.1. Deformation Response of Materials under Compression

**Figure 6:**
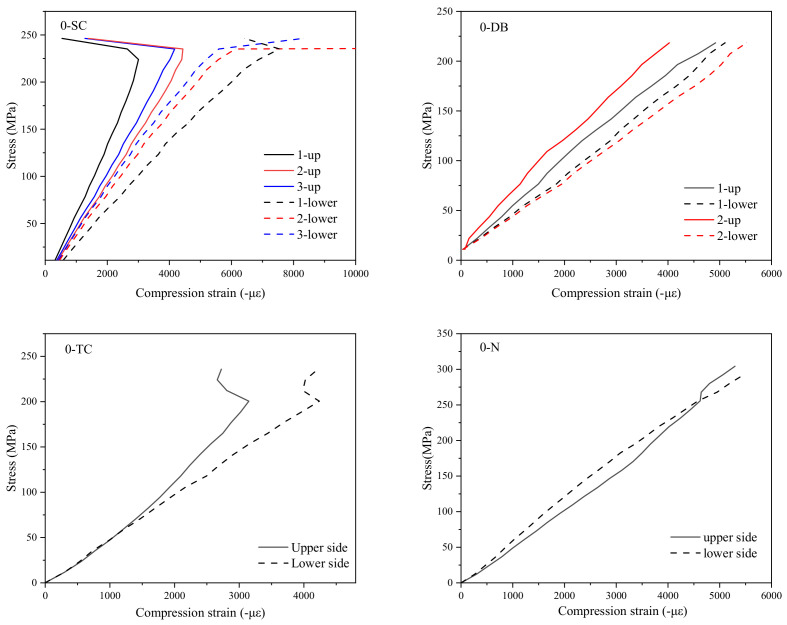
Stress–strain curves of specimens with particle density of 0 g/m^2^.

**Figure 7:**
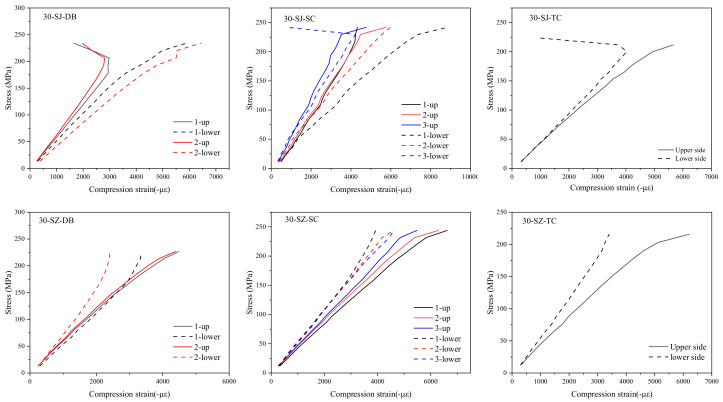
Stress–strain curves of specimens with particle density of 30 g/m^2^.

**Figure 8:**
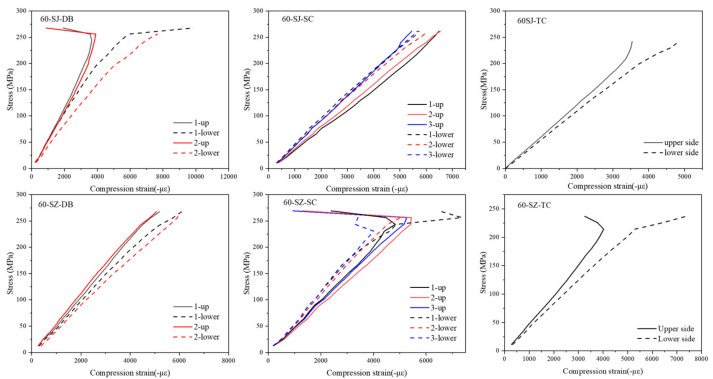
Stress–strain curves of specimens with particle density of 60 g/m^2^.

Figure 6, Figure 7 and Figure 8 show the stress–strain curves of specimens with a particle density of 0, 30, and 60 g/m^2^. Strains were measured by strain gauges. A pair of strain gauges was located on the same position of upper and lower surfaces of the specimen to monitor the possible bending deformation. By comparing the characteristics of stress–strain curves at different positions on the upper and lower sides, one can analyze the impact severity and mechanism of delamination defects on the mechanical properties of the laminated composite material.

It is seen that the stress–strain curves of the upper and lower sides of group 0-N specimens without a pre-delamination have a high degree of coincidence since the middle plane coincides with the neutral plane. In other words, when the specimen does not have a delamination defect, its compression deformation has good continuity and integrity and buckling does not occur. However, obvious differences exist in the stress–strain curves of upper and lower surfaces for the other three types of specimens, such as the No. 2 of the DB group, No. 1 of the SC, and the TC group. The difference between the stress–strain curves of the upper and lower surfaces when the strain gauges were located at the place of the delamination was the largest, such as the No. 1 strain gauges of the SC group. The farther the location of the strain gauge from the defect, the smaller the difference between the stress–strain curves of the upper and lower surfaces.

It was found that the difference between the stress–strain curves of the upper and lower surfaces of the 60-SZ-SC group and the 60-SZ-DB group was the smallest among several groups of specimens with the same defect type. On the contrary, the difference in the stress–strain curves of upper and lower surfaces was the largest for the specimens without interlayer toughening. This indicates that the interlayer properties have an appreciable influence on the delamination defects and thus affect the mechanical behavior of laminated composites. However, the stress–strain curves of the upper and lower surfaces of TC group were not significantly changed whether the particles were included or not, which preliminarily indicated that the particle toughening could not change the local deformation mode of TC group.

**Figure 9:**
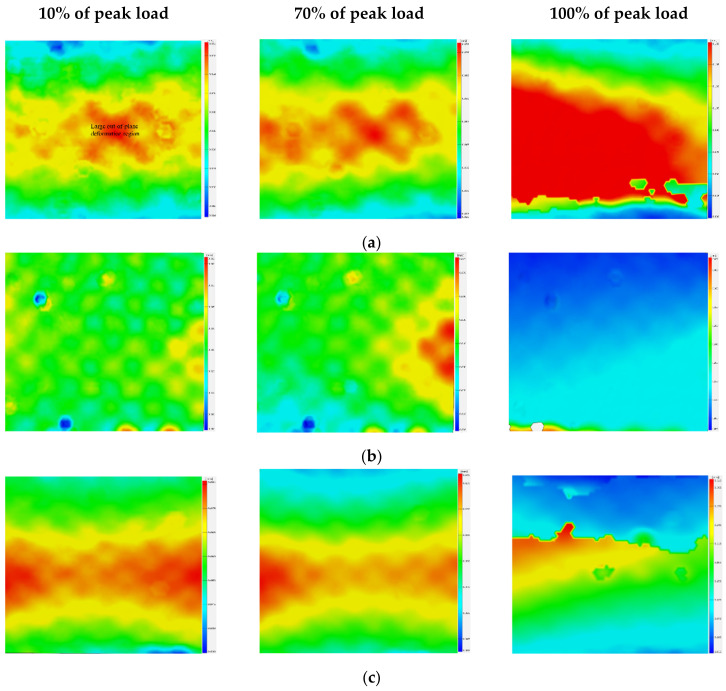
DIC diagrams of out-of-plane deformation field: (**a**) the DB group specimen, (**b**) the SC group specimen, and (**c**) the TC group specimen.

In order to further study the impact of delamination on the compression deformation of laminated composite materials, the out-of-plane displacement field during compression was monitored by using the DIC, as shown in Figure 9.

Through the whole process of monitoring of the out-of-plane displacement field by DIC, we found that a large out-of-plane deformation region existed, where the out-of-plane deformation was significantly larger than that of other intact positions of the specimen around the embedded defects for all types of specimens. The TC group specimen had a large out-of-plane deformation region at the initiation deformation stage. The large out-of-plane deformation regions of the DB group and the SC group specimens gradually expanded during the compression process, the possible reason might be that the internal delamination area of the two specimens was also gradually expanded during the compression process. Local buckling would make the material reach the deformation limit faster and reduce the compressive strength of the laminated specimens.

#### 3.1.2. Compression Damage Identification and Mechanical Response Analysis

**Figure 10:**
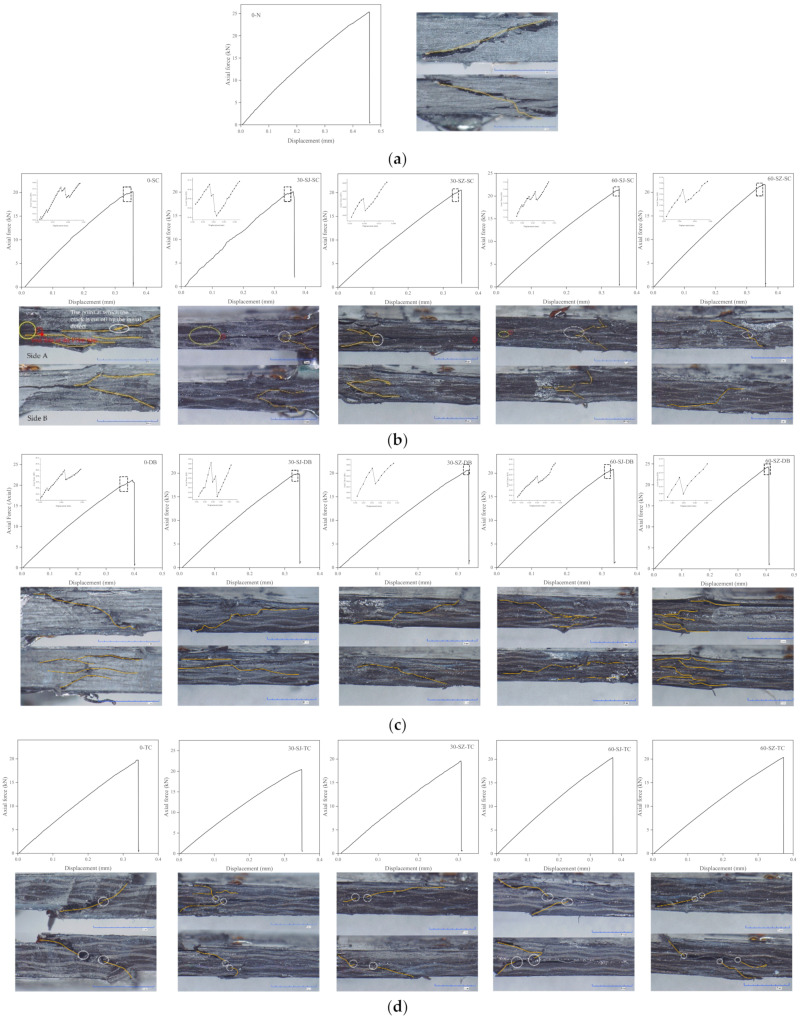
The load-displacement curves and damage morphologies of (**a**) the 0-N group, (**b**) the SC group, (**c**) the DB group, and (**d**) the TC group specimens.

The test results of the 0-N group in which the specimens were without any special treatment were used as the reference for analyzing the ones in other working conditions. Figure 10 shows the load-displacement curves and damage morphologies. As shown in Figure 10a, the variation of the load of the 0-N group with displacement was approximately linear. No obvious sudden change of load existed during the compressive process until the specimen is destroyed. The observation of the crack on the side of the specimen through the microscope after finishing the test shows that the crack propagated through the thickness continuously at a certain angle. Combined with the characteristics of the load-displacement curve, it is not difficult to infer that the failure of the 0-N group specimen was an instantaneous and brittle fracture.

Figure 10b shows the test results of the SC group specimen containing the surface crack. A sudden load descending in the load-displacement curves before the material reached the damage limit was seen whether the interlayer was toughened or not. This load descending did not cause the material to lose its load-bearing capacity immediately. Such a sudden load descending occured in the compression process of all specimens in the SC group and thus should not be caused by accidental test errors or noise. The load descending values of 0-SC and 30-SJ-SC specimens reached more than 100 N. With the increase of the particle density and magnetic guidance treatment, the load descending value decreased, and the failure load increased.

Since the location of the embedded defect is close to one side of the specimen, the side close to the embedded delamination of the specimen is called “side A” and the one away from the defect is called “side B” for convenience in discussion. Observing the damage morphology of side A shows that the embedded delamination of most specimens in the SC group can be seen after the test. The embedded delamination divided the specimen into two parts along the thickness direction, resulting in discontinuous cracks in the upper and lower parts or even completely different characteristics and trends of cracks. For example, the crack in the upper part of the 0-SC group in Figure 10b was a through thickness crack inclined at a certain angle, but it did not continue to expand downward when it contacted the embedded delamination, while in the lower half, the cracks were longitudinal splitting type, and there was more than one crack. From the damage morphologies on side A of the 0-SC, 30-SJ-SC, and 60-SJ-SC specimens, an obvious delamination propagation crack can be seen at the end of embedded delamination, as highlighted by the white oval mark in Figure 10b. However, such a crack is hard to observe on the specimens of the 30-SZ-SC group and the 60-SZ-SC group. Because of the delamination on side A, the damage on side B of most specimens also presents the characteristics of discontinuity.

Similar to the SC group, the specimen of the DB group also had a sudden load descending phenomenon during compressive loading, as shown in Figure 10c. Furthermore, the trend of the magnitude change of the load drop was similar to the one of the SC group. It further confirms that the perceptible sudden load descending phenomenon before complete failure should be related to the existence of some type of delamination defect. By comparing the damage morphologies on both sides, it can be seen that the crack characteristics on both sides are completely different, indicating that the cracks on the left and right sides were formed independently. Figure 9a shows that the out-of-plane displacement in the middle area of the DB group specimen was the most serious one at the beginning, so the initial damage was most likely to occur at this location, and the crack extended from the middle to both sides during the compressive loading. In this process, the left and right sides were independent of each other, so the cracks observed on both sides have different morphological characteristics.

From Figure 10d, it is seen that the results of the TC group specimens are quite different from the SC and DB groups. A sudden load descending did not exist in the load-displacement curves and all curves were similar to those of the 0-N group specimens shown in Figure 10a. Observing the damage morphologies on both sides of the TC group specimens shows that cracks on both sides are approximately symmetrical. Combined with the characteristics of load-displacement curves and symmetrically distributed damages on the upper and lower parts, one may speculate that the failure of the TC group specimen was instantaneous, similar to one of the 0-N group specimens. The cracks were separated into two parts by the embedded delamination and only the fibrous and matrix damages can be seen. Longitudinal growth of embedded delamination is not seen in the TC group, in contrast to the one in the SC group as marked by the yellow oval in Figure 10b. It is interesting to see that all cracks on the TC group specimens were connected with the embedded delamination, as noted by the white ellipse in Figure 10d. The possible reason is that the crack initiated from the embedded delamination boundary and extended outward along the thickness. Therefore, all cracks of the TC group specimens were connected with the embedded delamination. Furthermore, because the embedded delamination made the upper and lower parts of the specimen independent, neither were the crack starting points on the two sub-parts necessarily located in the same position, nor was the crack expansion necessarily in the same direction.

**Figure 11:**
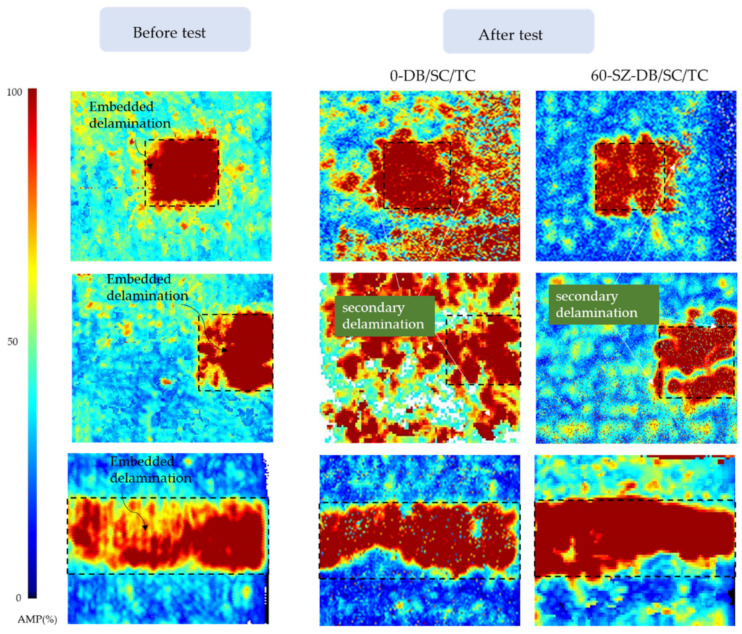
Delamination damage identification by C-scan.

Up to now, the compression failure mode of various specimens and the influence of different types of delamination defects on the compressive mechanical behavior of materials have been preliminarily analyzed. A sudden descending of load in the compression process of the DB group and the SC group specimens was observed. Previous experience showed that the energy release caused by the damage might lead to the sudden decline of the load. The more serious the damage was, the more intense the load decline was. However, observable surface damage was not seen before the complete failure of the specimen and only a popping sound similar to the one caused by the crack extension was heard during the compression test. Therefore, it is hypothesized that the sudden descending of load in the compression process of the DB group and the SC group specimens was due to the propagation of delamination defects. To verify this speculation, specimens containing different types of defects in the two groups with or without particles were selected for C-scanning. The results are shown in Figure 11.

To unify the expression, the area of delamination in the specimen after compression was called the final delamination area. The area of embedded delamination was called the initial delamination area. The part obtained by subtracting the latter from the former was called the secondary delamination.

Figure 11 shows that the delamination area after compression in the specimens of the 0-SC and the 0-DB groups greatly exceeded the initial area of the embedded delamination defects. The extra delamination areas were all connected with the ones of the initial delamination, indicating that the initial delamination propagated during the compressive process. Among those specimens embedded the same type of delamination, for the specimens with the large compression bearing capacity, such as the 60-SZ-DB and 60-SZ-SC, the delamination expansion was rather weak since the shape and range of the delamination after test were approximately the same as the initial embedded delamination. The delamination range of all specimens in TC group remained the same as the initial one. In other words, the delamination expansion did not occur during the compression process even for the specimen without particles filled into layers. Hence, one may conclude that delamination expansion causes the sudden load descending and the energy release due to the delamination expansion produces the popping sound heard in the test. The scanning results also show that the delamination growth direction of DB group specimens often extend laterally to the left and right sides, that is, perpendicular to the loading direction, while the delamination in SC group specimens will propagate along the loading direction in addition to transverse expansion.

#### 3.1.3. Comparison of Residual Strength of Materials

**Figure 12:**
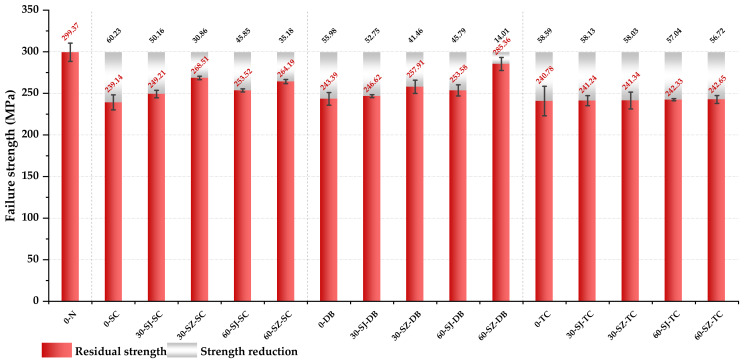
Comparison of residual compressive properties of different types of specimens.

It is seen that a great difference exists in the load response and the final failure form if the types of delamination defects contained in materials are different. In order to more intuitively reflect this difference, the residual compressive strength of materials of different kinds of specimens is compared, as shown Figure 12.

From Figure 12, it is seen that the average compressive strength of the laminated composites in group 0-N was the highest, reaching 299.37 MPa. The strength was significantly reduced if the laminated composites contained delamination defects between layers. In the case of no toughening, the ratio of strength reduction caused by three types of delamination damage was very close, and the strength reduction of the SC group was the largest and was 20.12% lower than that of the 0-N specimens, although the initial delamination area of the 0-TC group specimens was the largest. Referring to the delamination area comparison shown in Figure 11, the reason why the strength reduction caused by the three types of delamination was similar and the average strength of the 0-SC group was even lower than that of the 0-TC group is that the 0-DB group and the 0-SC group with smaller delamination area were more prone to delamination expansion before complete failure, resulting in their delamination area gradually approaching or even exceeding that of the 0-TC group.

It was also found that when the steel particles were used to toughen the interlayer of specimens in the SC group and the DB group, the improvement of the residual strength brought by the interlayer toughening was very significant. For the SC group, the residual strength of the 30-SZ-SC specimen was the largest and about 29.37 MPa higher than that of the 0-SC group, and the increase rate was close to 10.00%. The residual strength of the specimens in a 60-SZ-SC group increased by 25.05 MPa, which is slightly smaller than the one in the 30-SZ-SC group. When the particle density was the same, the residual strength of the specimens in the SZ specimen was often higher than that of the SJ specimen. It shows that the toughening with stainless steel particles can indeed improve the compressive residual properties of SC group specimens, and the magnetic guidance method can further improve this strengthening effect.

The strength of the specimens in the 0-DB group was 18.69% lower than that in the 0-N group, close to the proportion of strength reduction of the 0-SC and the 0-TC group. When a certain amount of stainless steel particles were added between layers and guided by the magnetic (specimens in DB group), the residual strength increased very significantly. The average strength of the specimens in the 60-SZ-DB group reached 285.36 MPa, only 4.77% lower than that of the 0-N group.

However, the interlaminar toughening by adding stainless steel particles had little effect on the improvement of compressive strength for the TC specimens, and the difference between the largest and smallest strength was less than 2.00%.

Based on the comparison and analysis, one may conclude that the initial delamination area in the laminated composite cannot be directly used as evidence to judge the residual compressive strength since delamination propagation may occur during the compressive process. The final delamination area should be used as evidence to judge the residual strength of laminated composites.

### 3.2. Damage Mechanism

#### 3.2.1. Relationship between Fracture Toughness and Residual Strength after Delamination

In this section, the reason the in-plane compression strength can be improved by increasing the interlaminar toughness is given. First of all, the enhancement of the material properties by the particles themselves should be excluded, because the particles are only located between ply-3 and ply-4, which is not enough to cause a large difference in properties from the perspective of volume content. Moreover, if it is due to the in-plane reinforcement of the particles, the strength of SJ group should be higher than that of SZ group, because randomly distributed particles can enhance the compression axis better, but the SZ group should be reinforced in the thickness direction, which is inconsistent with the test results.

**Figure 13:**
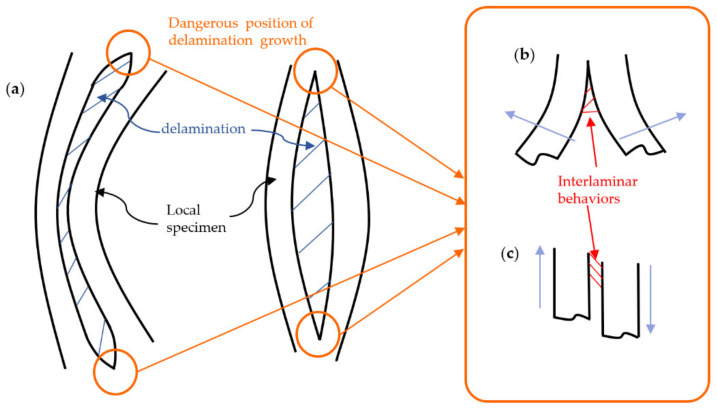
Schematic diagram of the propagation of delamination defect: (**a**) different buckling modes at delamination position, (**b**) crack opening, and (**c**) dislocation.

In addition, according to the damage morphology of the specimens and the internal C-scan images, one can find that some specimens have the propagation of delamination from its initial state (embedded PTFE film) in the compression process, such as specimens in SC group and DB group. From Figure 6, Figure 7, Figure 8 and Figure 9, one can speculate that the propagation of delamination should be caused by the local buckling due to the initial delamination defect. The embedded film divides one specimen into two parts along the thickness locally. Therefore, once buckling occurs, it is difficult to ensure the continuity of the deformation of the upper and lower parts, in other words, the deflections of them are different, resulting in the opening and dislocation shear at the edge of the delamination, as shown in Figure 13, which may further lead to the mode I and mode II propagation of the existing cracks.

Therefore, the interlaminar fracture toughness has become the most important factor affecting the delamination growth. The enhancement of toughness would make the crack growth more difficult, and thus limit the expansion of the defect range and reduce the final impact of delamination on the compressive properties of the laminated composites.

**Figure 14:**
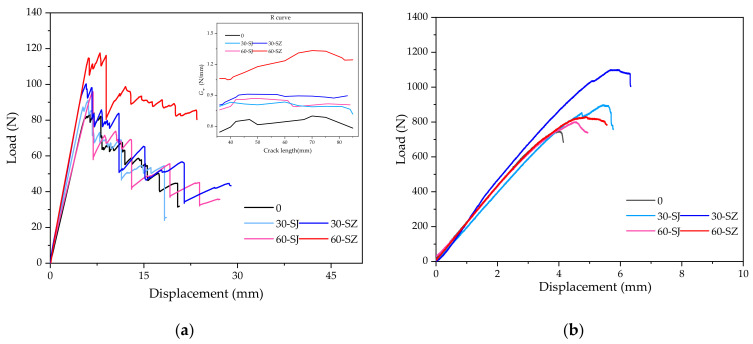
Load-displacement curves of specimens by (**a**) DCB tests, (**b**) ENF tests.

As shown in Figure 14 and Table 1, the interlaminar fracture toughness test results under various working conditions are used as an important reference for studying the relationship between interlaminar fracture toughness and compression performance. The symbols used for naming specimen refer to the same meaning as above.

It can be seen that the toughening method can effectively improve the interlaminar fracture toughness of materials. Among them, the interlaminar mode II fracture toughness of the 30-SZ group increased by 87.72%, which is significantly higher than that of the other groups and reached twice that of the SJ group specimens with the same particle density. In addition, for mode I fracture toughness, the enhancement rate of the 60-SZ group was the highest, reaching 81.88%, while the enhancement rate of the 30-SZ was the highest in other working conditions, but it was still less than 30.00%. In general, under the same particle density, the fracture toughness enhancement rate of the SZ group was much higher than that of the SJ group, which indicates that magnetic guidance, as an auxiliary means of particle arrangement direction, plays a very significant role in improving the interlaminar fracture toughness.

It is seen that the mode II fracture toughness of the 30-SZ group and the mode I fracture toughness of the 60-SZ group were increased by more than 80%, much higher than the corresponding ones of other types of specimens, while the mode I fracture of the 30-SZ group and the mode II fracture toughness of the 60-SZ group were much lower. The results may be helpful to identify whether a certain type of delamination defect was affected by mode I or mode II fracture toughness.

The covariance (*COV*) was introduced to measure the correlation between the residual compressive strength and mode I/II fracture toughness for different types of specimens. Test results are listed in Table 2.

Through the comparison of values of *COV*, it is seen that most of the residual strength of the laminates is positively correlated with the mode I and mode II interlaminar fracture toughness. This indicates that mode I and mode II fracture toughness affected the residual compressive strength of the material simultaneously. For specimens of the DB group, the correlation between the residual strength and the mode I fracture toughness is stronger than that with the mode II fracture toughness, indicating that the mode I fracture toughness was the main factor affecting the residual strength of the specimens of DB group. For specimens of the SC group, the residual strength is more related to mode II fracture toughness. Therefore, the secondary growth of the delamination in the DB group was mostly caused by the crack opening during the compression process, while the one in the SC group was mostly caused by the shear at the edge of the delamination.

In addition, the *COV* values of the TC group are small, indicating that the fracture toughness affects the residual strength of specimens of the TC group little. This can be inferred from the C-scan images shown in Figure 11. It is seen that the delamination defects contained in the TC group specimens without toughening do not produce significant secondary growth during compression and thus the interlayer toughening is of no significance for such cases.

#### 3.2.2. Qualitative Dynamic Damage Relationship Description

It is seen that not the initial, but the final state of delamination damage is directly related to the residual compressive properties of the delaminated composites. Therefore, whether the laminates have a secondary growth of delamination or not becomes the key to determine the residual properties of materials. Although the reason for the secondary growth of delamination is complex, the macro stress state is simple under uniaxial compression. A variable *D*, called delamination limit load, was introduced to describe the relationship between delamination expansion and compression load. When the applied load on the specimen was greater than or equal to the *D*, the delamination began to propagate.

Previous studies showed that a negative correlation between the delamination area and the compression failure limit load existed; that is, the larger the delamination area, the lower the compression failure load of the material [46]. Therefore, it is only necessary to express the changing trend of *F_s_* with the lager delamination area through a monotonically decreasing curve. It should be noted that the delamination area refers to the final delamination area.

**Figure 15:**
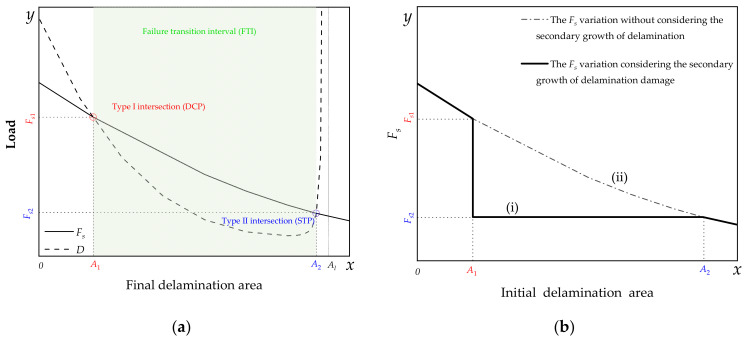
Schematic diagram of dynamic damage relationship during compression: (**a**) *F_s_*—final delamination area curves, (**b**) *F_s_*—initial delamination area curves.

Based on the test results and assumptions, a qualitative dynamic damage relationship is proposed to describe the dynamic process of strength decline of laminated composites caused by the continuous expansion of delamination damage during compression. The dynamic damage relationship is schematically described in Figure 15 by curves. Figure 15a shows the relationship between the *F_s_* and the final delamination area, including the one without an initial delamination such as the specimen in the 0-N group. Figure 15b shows the relationship between the *F_s_* and the initial delamination area.

When the final delamination area is close to 0, the delamination limit load *D* should be greater than the compression failure load *F_s_*. However, delamination propagation may occur before failure if the interlayer performance is relatively poor for some other material and thus it is also possible that *D* is smaller than *F_s_*. Overall, *D* should not be an infinite value at *x* = 0, which means that the *D* curve has a *y*-intercept. In addition, the test results also show that when the initial delamination area is less than a certain value, secondary growth of the initial delamination damage occurs before the compression damage. This suggests that when the delamination area is within a certain range, delamination propagation occurs before the damage, which means that *F_s_* is greater than *D* in this case. At the same time, the test results of the TC group also show that once the delamination area reaches a certain value, the delamination defect can no longer propagate, which means that *D* gradually becomes larger than *F_s_*.

At the limit state, i.e., the range of delamination almost covers the whole specimen, the delamination growth cannot occur and thus the value of *D* should tend to positive infinity. Based on the above discussion, combined with the assumption that *F_s_* is monotonically decreasing, a simplified qualitative D-final delamination area curve that is first monotonically decreasing and then monotonically increasing to infinity can be obtained, as shown in Figure 15a.

There are two intersection points in Figure 15a. The one at *x* = *A*_1_ is called type I and the one at *x* = *A*_2_ is called type II. Delamination growth occurs when the area of the initial delamination defect is greater than *A*_1_ and less than *A*_2_, so the residual performance of the material is not directly reflected by the initial delamination area. Delamination propagation releases energy and thus reduces the load. The load rebounds when it is less than D. If the delamination area value is still within the above interval, i.e., *A*_1_ < *x* < *A*_2_, the process of load drop and rebound continues with the delamination propagation until *D* > *F_s_*. Therefore, once the delamination defect area of the material surpasses *A*_1_, even a little, the material performance would rapidly decline from *F_s_*_1_ to *F_s_*_2_, as shown in Figure 15b; hence, type I point is named as “dangerous critical point” (DCP). In the interval *A*_1_ < *x* < *A*_2_, the material can still bear load but gradually approaches the failure point. Therefore, such interval is named the “failure transition interval” (FTI). Furthermore, the residual strength *F_s_*_2_ corresponding to type II point determines the final residual performance of all materials whose initial delamination area values are within the FTI, and thus type II point is named as the “strength target point” (STP).

From the above discussion, to effectively improve the delamination damage tolerance of the material, the delamination area corresponding to DCP should be as large as possible and the one corresponding to STP should be reduced to as small as possible. This can not only reduce the probability that the delamination area value is just located in the FTI (because once the delamination damage area value is just in this interval, the secondary growth is inevitable), but also ensure less strength reduction even if the secondary growth of delamination is inevitable.

From the characteristics of the curves, the shorter the FTI or the closer the curve (i) in Figure 15b to curve (ii) is, the higher the delamination damage tolerance of the material. If the two curves in Figure 15a are tangent or without intersection, the most ideal state, DCP, and STP are combined into one or do not even exist at all, and the FTI length is zero in such a case, the value of *D* is always larger than the *F_s_*, and delamination does not propagate during the compression. However, in this case, interlayer toughening is not effective in improving residual strength. This is similar to the TC group tests because the initial delamination areas of the TC group specimens are already larger than the area value *A*_2_ corresponding to the STP, which means that delamination does not precede compression damage. Therefore, the increase in interlaminar toughness in this case does not affect the compression strength of this class of materials.

However, for specimens with suitable delamination areas, such as the DB and SC groups, the interlayer toughening method can effectively improve the residual properties of materials. In other words, the toughening of the interlayer can change the characteristics of the *D* curve, change the intersection position, and enhance the residual performance. Furthermore, considering that *D* should be a function influenced by multiple variables, other conditions may affect the characteristics of the curve, such as the position of the delamination defects in the thickness direction, the number of delamination defects, and the geometry of the delamination.

## 4. Conclusions

Carbon fiber laminates with three types of delamination defects, i.e., the deeply buried delamination, the through-width delamination, and the surface delamination, were successfully manufactured to experimentally study the relationship between the residual compressive properties and the interlayer toughness, as well as the type of delamination defects. A new magnetically guided stainless steel particle interlayer toughening method was used to enhance the interlayer toughness. A dynamic damage relationship was proposed based on the test results. The experimental results showed that the surface delamination reduced the compressive strength of the laminates most with 20% reduction, while the percentage reduction of the compressive strength of laminates with deeply buried delamination and through-width delamination was slightly lower than that of the surface delamination.

The improvement of interlaminar toughening can effectively improve the residual compressive properties of some laminated plates containing delamination. Improving the mode I fracture toughness can improve the residual strength of the specimens containing the embedded delamination. The strength reduction decreased from 55.98 MPa to 14.01 MPa when the mode I fracture toughness was increased by 81.88%. Improving the mode II fracture toughness can enhance the residual strength of the specimens with the surface delamination. The strength reduction decreased from 60.23 MPa to 30.86 MPa when the mode II fracture toughness was increased by 87.72%.

In addition, it was found that generally the property degradation caused by secondary delamination should be considered, but if the delamination reached a certain level, the property degradation caused by the secondary delamination growth was negligible since the delamination area did not increase anymore, as evidenced by the test results of the TC group. The significant increase in residual strength in the DB and SC groups indicated that increasing the fracture toughness to prevent secondary delamination was an effective means of increasing the delamination damage tolerance of the laminate. A magnetically guided stainless steel particles interlayer toughening method could effectively inhibit delamination growth and thus improve the residual properties of laminates.

## Figures and Tables

**Table 1:** Interlaminar fracture toughness test results and enhancement rates compared with the specimen without particles.

Specimen	*G_IC_*/(N/mm)	Enhancement Rate/%	*G_IIC_*/(N/mm)	Enhancement Rate/%
0	0.67	0	1.91	0
30-SJ	0.76	13.43	2.64	38.22
30-SZ	0.84	25.37	3.59	87.72
60-SJ	0.79	17.91	2.31	20.94
60-SZ	1.21	81.88	2.52	31.94

**Table 2:** *COV* between fracture toughness and residual strength.

	*G_IC_*	*G_IIC_*
DB	2.76	1.726
SC	1.33	4.76
TC	0.10	0

## Data Availability

The data used to support the findings of this study are included within the article and are also available from the corresponding author upon request.
